# Comparative Evaluation of Bone Marrow Aspirate with Trephine Biopsy in Hematological Disorders and Determination of Optimum Trephine Length in Lymphoma Infiltration

**DOI:** 10.4084/MJHID.2014.002

**Published:** 2014-01-02

**Authors:** Surbhi Goyal, Usha Rani Singh, Usha Rusia

**Affiliations:** Department of Pathology, University College of Medical Sciences, Dilshad Garden, Delhi, India

## Abstract

**Introduction:**

Bone marrow examination is an indispensable diagnostic tool to evaluate neoplastic and non neoplastic hematological diseases.

**Aims:**

To compare bone marrow aspirate with trephine biopsy in hematological disorders. To determine the optimum trephine preprocessing length in lymphoma infiltration.

**Methods:**

Diagnostic comparison was done between simultaneous bone marrow aspirates and trephine biopsies in 449 patients. Biopsies were fixed in formalin, decalcified in 5.5% EDTA and routinely processed. Concordance rates and validity parameters for aspirate were calculated. Three deeper sections of trephine biopsy, cut at 0.1–0.2 mm intervals, were assessed for lymphoma involvement. Proportion of biopsies showing marrow infiltration by lymphoma cells was plotted against trephine length and correlation was assessed.

**Results:**

Aspirate had a high sensitivity for acute leukemia (89.4%) and multiple myeloma (88.5%), moderate for NHL (67.6%) and nonhematopoietic metastases (58.3%) and low for aplastic anemia (38.5%) and Hodgkin lymphoma (5%). Aspirate has no role in granulomatous myelitis and myelofibrosis. Lymphoma positivity increased with trephine length, with maximum positivity (68.9%) seen in 17–20 mm group and no further gain beyond 20 mm. (lymphoma positivity ≤16mm=40.3% and ≥17mm=66.1%, p=0.0011).

**Conclusion:**

Aspirate has a high specificity; its sensitivity depends upon the type of disease. Apart from few conditions, in which aspirate alone is sufficient, biopsy is mandatory in most. Preprocessing trephine length of 17–20 mm examined at multiple deeper levels was found optimal for assessing lymphoma positivity.

## Introduction

Bone marrow examination is an indispensable diagnostic tool in the evaluation of various hematological disorders, non hematological malignancies, pyrexia of unknown origin and infective diseases.^1^ It is also valuable for follow up of patients undergoing chemotherapy and bone marrow transplantation.^1^,^2^ Involvement of marrow by metastases has a significant impact on patient management and prognosis.^3^ Bone marrow examination serves to establish or confirm a primary diagnosis of lymphoma or to determine the extent of disease dissemination for staging purposes.^4^ Rarely, bone marrow examination has been useful in detecting non-hematopoietic malignancy in clinically unsuspected cases.^5^ At times, marrow metastases may have normal serum chemistry and hematologic parameters and may even be missed by bone scans and advanced imaging modalities.^6^ This fact highlights the importance of using sensitive techniques for the detection of marrow metastasis. Accurate diagnosis of myelitis by disseminated infections is important for timely management.

Bone marrow aspiration (BMA) is a simple, reliable and rapid method of marrow evaluation. Trephine biopsy provides more comprehensive information regarding the marrow cellularity, architectural patterns and overall hematopoiesis. But biopsy is a painful procedure and its processing takes at least 48–72 hours. So, to perform trephine biopsies in all patients may not be cost effective in terms of clinician and laboratory personnel time, efforts and patient discomfort. Few studies have analyzed the diagnostic accuracy of bone marrow aspirate in comparison with trephine biopsy.^7^–^10^ Literature on correlation of lymphoma positivity with trephine biopsy length is even sparse.^11^,^12^ With these considerations, a prospective study was conducted with the objectives of comparing the accuracy of BMA with trephine biopsy done simultaneously in the diagnosis of hematological disorders and to determine the optimum trephine preprocessing length to assess lymphoma infiltration.

## Material and Methods

This single institution prospective study was approved by the Institutional Ethics Committee and informed consent was obtained from all the patients.

### Subject population

**Figure 1:**
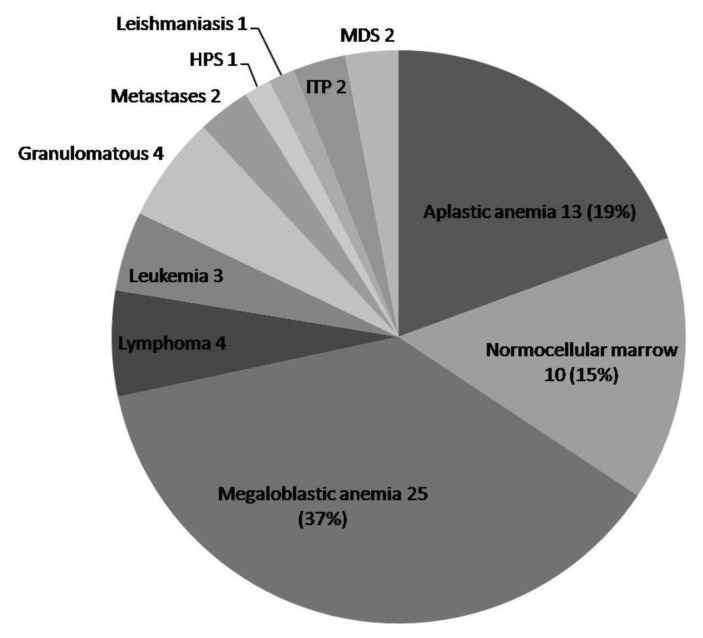
Pie chart showing distribution of patients presenting with pancytopenia. The number(s) following diagnosis represent actual number of patients, while number(s) in parenthesis represent the percentage of pancytopenic patients.

From January 2011 to February 2012, 514 patients were recruited in the study and underwent both bone marrow aspirate and biopsy simultaneously. Of these, 65 (12.6%) biopsies were inadequate for assessment and were excluded from analysis. So, final study cohort comprised 449 patients who had undergone both aspirate and biopsy simultaneously. Patient demographic information, clinical history including physical findings, chemo/radiotherapy, complete blood count with peripheral smear findings and indication of bone marrow were collected by one author. Aspirate findings were compared to that of trephine biopsy. Of these, 382 patients were diagnosed or follow up cases of hematolymphoid malignancy and their distribution is shown in table 1. Sixty seven patients presented with pancytopenia/bicytopenia and bone marrow was done to detect the etiology (Figure 1).

### Bone marrow aspirate and trephine biopsy

BMA was done using Salah’s needle and 0.25 to 0.5 ml of aspirate was withdrawn with a 20 ml plastic syringe from posterior superior iliac spine. Aspirated material was delivered onto clean glass slides and smears were prepared immediately. After that, trephine biopsy was performed using Jamshidi’s needle through the same incision, approximately 0.5–1 cm away from the site of aspiration to avoid obtaining a hemorrhagic biopsy. Peripheral smears and marrow aspirate smears were stained by Wright’s stain. Trephine biopsies fixed in 10% neutral buffered formalin, were subjected to decalcification in 5.5% EDTA solution for 24 hrs. After decalcification, the preprocessing length of trephine biopsy was measured with a metric scale and biopsy was routinely processed in automated tissue processor and embedded in paraffin blocks. In cases where a trephine was in several pieces, the total length was recorded. 2–3μm thick sections were cut and stained with hematoxylin & eosin. All the smears and sections were reviewed by two experienced pathologists in consensus. At least three deeper sections, cut at intervals of 0.1–0.2 mm, were examined to assess marrow involvement in patients of lymphoma and suspected metastases. While examining the aspirate, the pathologists were blinded to biopsy findings. BMA and biopsy findings were compared. Wherever indicated, histochemistry was performed. Gömöri’s reticulin and Masson’s trichrome were performed to grade marrow fibrosis according to European Consensus grading system.^13^ In cases where tuberculosis was suspected, cold Ziehl Neelson was performed to stain for acid fast bacilli (AFB). PAS was done to look for glycogen and fungal hyphae. For immunohistochemistry, sections on poly-lysine slides were taken and immunohistochemistry was done by standard streptavidin biotinylated peroxidise method. In suspicious cases of marrow infiltration by lymphoma, panel of antibodies (CD45, CD 20, CD 15, CD 30, CD 3, CD 5) was employed for confirmation and further subtyping. Antibodies against λ and κ light chains were used to establish the monoclonality in neoplastic plasma cells. To confirm nonhematopoietic marrow metastases in suspicious cases, antibodies against cytokeratin, Neuron specific enolase, CD99, S-100 and epithelial membrane antigen were used wherever required.

### Statistical analysis

Results were statistically analyzed using SPSS software (version 17.0, SPSS, Chicago, Illinois, USA). Concordance rates were calculated between aspirate and biopsy. In groups with sufficient sample size, validity parameters were calculated. Proportion of trephine biopsies showing lymphoma infiltration was plotted on y axis and total preprocessing trephine length on x axis, in increments of 4 mm. Fischer’s exact test was used to analyze the significance in lymphoma positivity between two groups and *P* value <0.05 was considered statistically significant.

## Results

### Lymphoma

**Figure 2:**
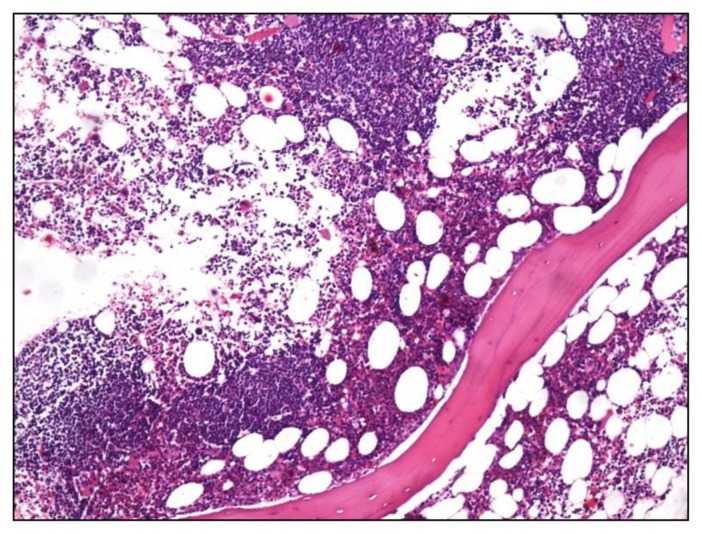
Trephine biopsy shows nodular, paratrabecular infiltration by atypical lymphoid cells in a patient of small lymphocytic lymphoma (H&Ex100).

Lymphomas accounted for 40.1% of all patients. Of these, 73.9% (133/180) patients were of Non Hodgkin Lymphoma (NHL) and Hodgkin lymphoma comprised 26.1%. Of 133 trephines being evaluated for NHL staging, 67 (50.4%) showed marrow infiltration in the form of paratrabecular nodular, interstitial or diffuse pattern (Figure 2). Of these, 20 were SLL/CLL, 15 were follicular and mantle cell, 13 were large cell type and rest could not be further subtyped. 46 out of these 67 aspirates were reported as positive for lymphoma infiltration, 15 were reported as negative and six were inadequate. In 66 cases, both biopsy and aspirate were negative for lymphoma infiltration. Chemotherapy induced changes comprised increased vessel density, necrosis and marked fibrosis of intertrabecular space. These were seen in four trephine biopsies but not in aspirates.

**Figure 3:**
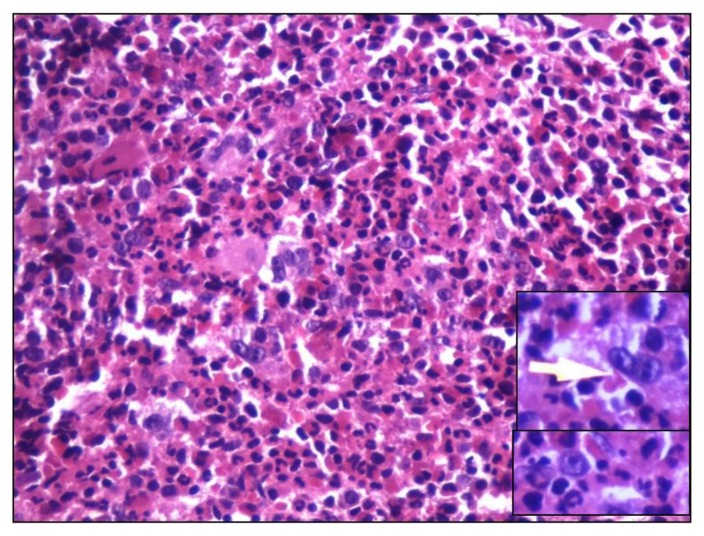
High magnification of trephine biopsy shows classical binucleate Reed Sternberg cells (Inset with arrow) and mononuclear variant Reed Sternberg cells (Inset) in a polymorphous background comprising of plasma cells, eosinophils and lymphocytes (H&Ex400).

In Hodgkin lymphoma, marrow involvement was seen in 42.5% (20/47) patients. Both biopsy and aspirate were negative for lymphoma in rest 27 patients. Large binucleate cells with moderate amount of cytoplasm, vesicular nucleus and prominent eosinophilic nucleolus (classical Reed Sternberg cells) and mononuclear cells (variant RS) were seen (Figure 3) and confirmed by bright paranuclear positivity for CD 15/ CD30. Focal fibrosis and necrosis was seen in 12 cases. Epithelioid cell granulomas were found in five of them. However, stain for AFB was negative. Only 1 out of 20 aspirate (5%) showed few large atypical mononuclear variants and occasional classical RS cells, suggestive of marrow involvement. 17 aspirates (85%) were reported as negative for marrow infiltration.

### Chronic myeloproliferative neoplasm (CMPN)

**Figure 4:**
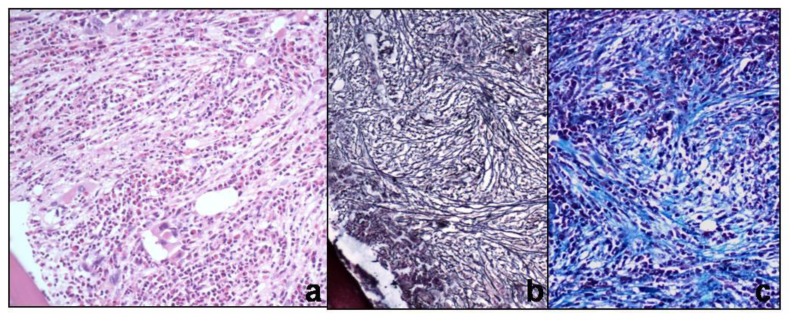
a) Trephine biopsy of Primary myelofibrosis showing cellular marrow with preponderance of myeloid precursors, atypical megakaryocytes and fibrosis (H&Ex200), b) Gomori’s reticulin shows grade 3 fibrosis (Reticulin ×200) c) Fibrosis is confirmed by bluestained collagen (Masson trichrome ×200)

Of 81 patients, 70 were of chronic myeloid leukemia (CML), 9 of primary myelofibrosis (PMF) and 2 of hypereosinophilic syndrome (HES). 28 patients of CML in chronic phase (CP) had minimal fibrosis on biopsy. 30 CML-CP biopsies showed increased number of micromegakaryocytes and grade 2 reticulin fibrosis. 7 of these (23.3%) yielded inadequate aspirates. Trephine biopsy and aspirate were suggestive of blast crises in 10 patients. In 2 patients, peripheral smear and aspirate showed blasts less than 10% suggestive of chronic phase, but trephine biopsy showed focal aggregates of blasts in an entire intertrabecular space, warranting a diagnosis of blast crisis. 9 patients of PMF had cellular marrow with grade 3 reticulin fibrosis and collagenisation (Figure 4). Clusters of atypical megakaryocytes having hyperchromatic bulbous nuclei, were seen adjacent to vascular sinuses. BMA showed dry tap in 7 of these cases after repeated attempts and cellular marrow particles having atypical bizarre megakaryocytes in the remaining 2 cases. Aspirate and biopsy were in agreement in two HES patients.

### Acute leukemia

BMA was in agreement with biopsy in 39 patients, but was inadequate in 5 patients who had tightly packed marrow with blasts on biopsy. Nine patients were in complete hematological remission both on aspiration and biopsy.

### Metastases

**Figure 5:**
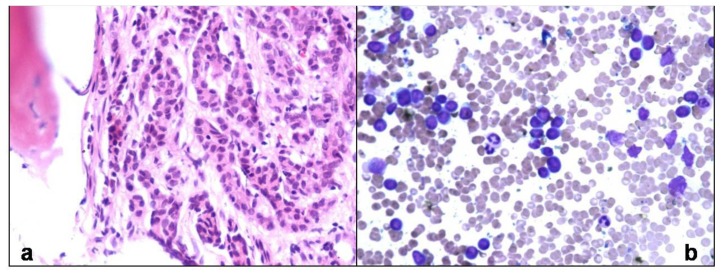
a) Trephine biopsy shows metastatic neuroblastoma in a child (H&Ex 400) b) Bone marrow aspirate of the same patient shows metastatic small round cell tumor (Wright stain ×400).

Definitive evidence of marrow metastases was seen in 10/23 patients. Small round cell tumors - Ewing’s/ PNET, neuroblastoma, Wilm’s tumor and retinoblastoma were the primary in children (Figure 5).

Six adult patients (mean age=59yrs) presented with backache, anemia and had multiple lytic lesions in vertebral column with differential of metastases and multiple myeloma. Bone marrow examination revealed metastatic adenocarcinoma from prostate, breast, gastrointestinal tract and lung. Of ten marrow metastases, aspirate detected only six. Three aspirates were negative and one was inadequate. In rest 13 suspected patients, both aspirate and biopsy were negative for metastases.

### Multiple myeloma

**Figure 6:**
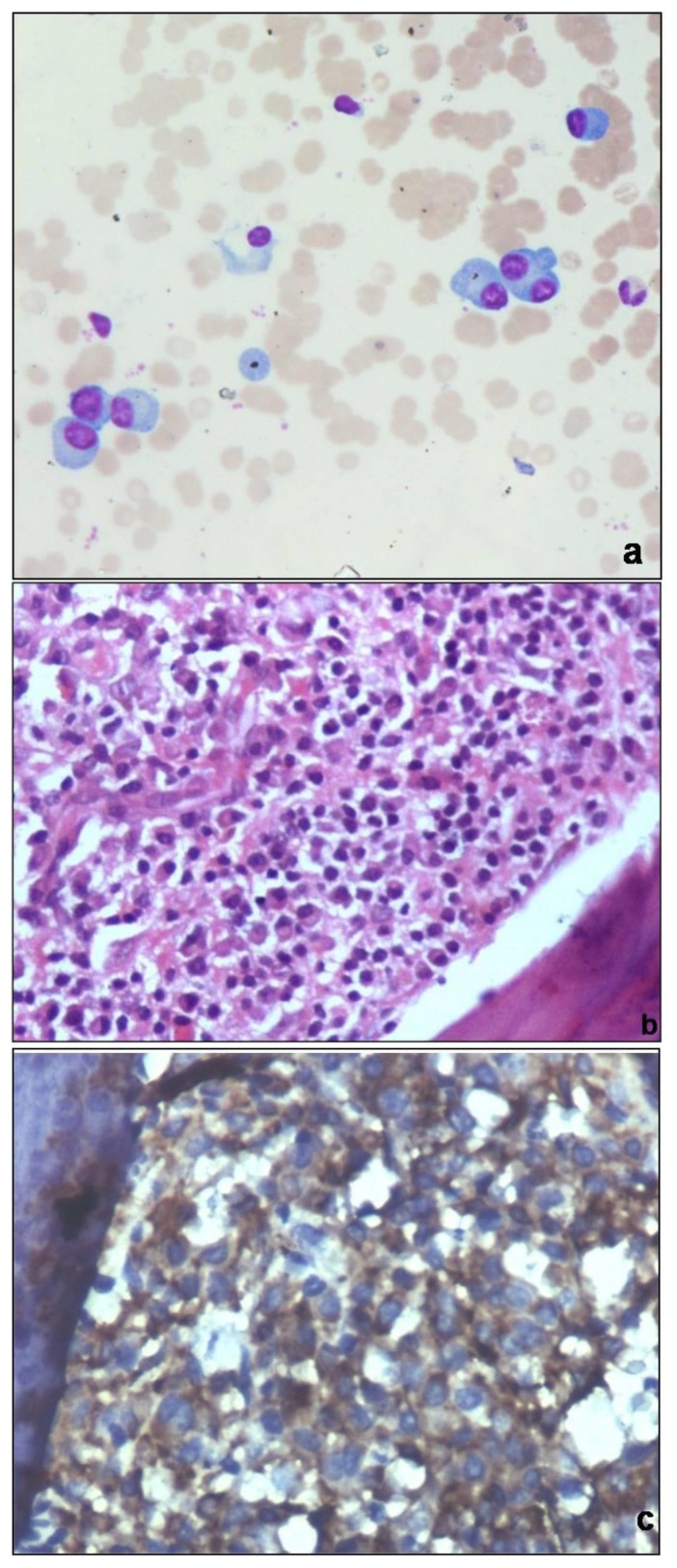
a) Bone marrow aspirate shows diluted marrow with scattered plasma cells in a patient of multiple myeloma, overall percentage 10% (Wright stain × 400) b) Biopsy from same patient shows paratrabecular collection of plasma cells and plasmablasts (H&Ex400) c) On immunohistochemistry, these cells show λ light chain restriction, confirming the monoclonality (Immunostain λ ×400).

Biopsy and aspirate were concordant in 23/26 (88.5%) patients of multiple myeloma. Three aspirates were hypocellular due to fibrosis with focal aggregation of myeloma cells on biopsy sections. On immunohistochemistry, these cells showed evidence of monoclonality by κ or λ light chain restriction (Figure 6). Biopsy and aspirate did not show plasmacytosis in rest 5 patients.

### Pancytopenia/bicytopenia

**Figure 7:**
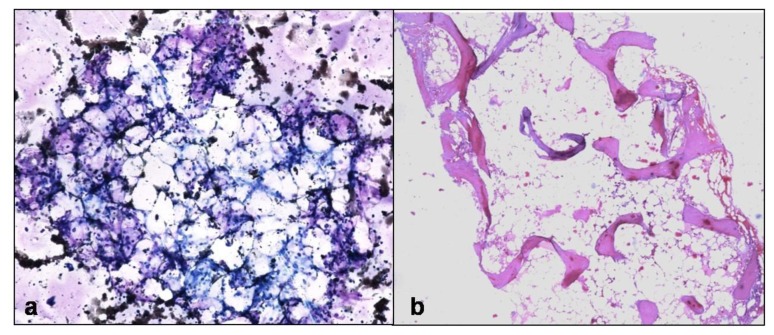
a) Bone marrow aspirate from a pancytopenic patient showing hypocellular marrow particles with entangled lymphocytes and plasma cells and occasional erythroid precursors (Wright stain × 400) b) Biopsy shows markedly hypocellular marrow with increased fat spaces, confirming the diagnosis of aplastic anemia (H&E × 100)

In 67 pancytopenic patients, hypercellular marrow was seen in 44, hypocellular marrow in 13 (Figure 7), and marrow was normocellular in 10 patients. Figure 1 shows the spectrum of cases presenting with pancytopenia.

Marrow metastases from neuroblastoma and small cell carcinoma along with secondary myelofibrosis on biopsy presented as pancytopenia, for which marrow was done.

### Comparison of bone marrow aspirate with trephine biopsy

Concordance rates were calculated between BMA and trephine biopsy (Table 2). In larger subgroups we also calculated validity parameters taking trephine biopsy as gold standard (Table 3).

### Correlation of trephine length with lymphoma positivity

**Figure 8:**
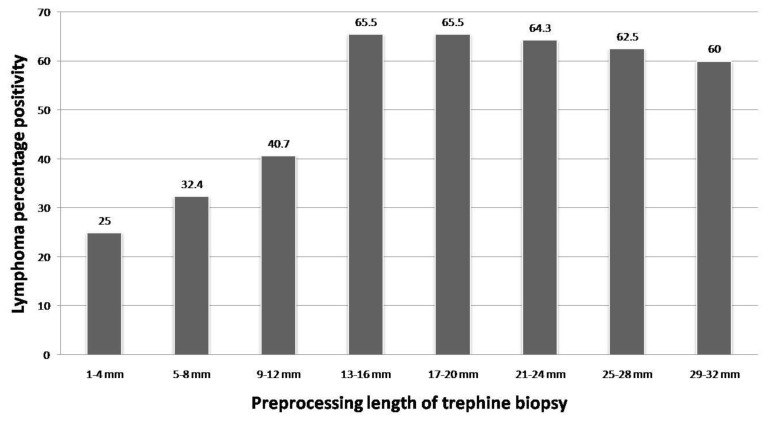
Bar graph depicting the percentage of lymphoma positivity in bone marrow, in relation to preprocessing trephine biopsy length.

We had 184 adequate biopsies for lymphoma staging in our study. Of these 49.4% (91) were positive for lymphoma infiltration after examining three sections at deeper levels. The mean length of trephine core was 14 mm, ranging from 1–32 mm. We found that lymphoma positivity showed a rising trend with length of trephine core, with maximum positivity (68.9%) seen in 17–20 mm group, but no further improvement beyond 20 mm (Table 4, Figure 8). Based on this two groups were made, taking 16mm as cut-off. Fischer’s exact test was applied and the difference in both the groups was found to be statistically significant (lymphoma positivity ≤16mm=40.3% and ≥17mm=66.1%, p=0.0011).

## Discussion

We have evaluated the role of bone marrow aspirate in comparison with trephine biopsy in diagnosis of various hematological disorders. In 28.2% cases aspirate was nondiagnostic, with an overall sensitivity of 71.8%. Jamshidi and Swain reported that in 14–16% patients, aspirate was non diagnostic.^14^ Immunohistochemistry was diagnostically helpful in our study in case of NHL, Hodgkin lymphoma and multiple myeloma, where equivocal morphology and low tumor cell burden posed a dilemma. In biopsies with few suspicious cells or crush artifact, it can increase the diagnostic accuracy by unmasking the obscured patterns and morphology.

We found bone marrow aspirate to be 100% specific in most of the disorders, but sensitivity and accuracy depends upon the disease being evaluated. In hematological malignancies, highest sensitivity was seen in acute leukemias (90%), multiple myeloma (88.5%) followed by CMPNs (77.2%). In diffuse marrow pathologies like nutritional anemia, leishmaniasis, ITP and HPS, diagnostic sensitivity of marrow aspirate was 100%. Trephine biopsy did not provide any additional information. Therefore, aspirate may obviate the need of biopsy in such situations.

Frequency of positive BMA in metastatic marrow varies from 23% to 100% in different studies.^15^–^18^ In our study, 41.7% of aspirates missed marrow metastases, similar to results of previous studies. Focal deposit of nonhematopoietic malignant cells and tumor associated desmoplasia, necrosis are the cause of dry tap on aspiration. According to Chandra et al, aspirate along with imprint smear has similar diagnostic accuracy to trephine biopsy and can avoid the inevitable delay caused by decalcification and routine histopathological processing of the biopsy.^7^

Overall incidence of marrow involvement by Hodgkin and Non Hodgkin lymphoma was quite high (42.5% and 51.8% respectively) in our study. Various studies have reported marrow infiltration in lymphoma ranging from 27.1 to 55.1%.^19^ This variation can be attributed to higher incidence of Hodgkin lymphoma in our population and inclusion of different proportion of patients of early/advanced stage. Only two third of NHL positive marrows were picked up on aspirate and 23.9% were missed on aspiration. BM biopsy renders information which cannot be determined from aspiration, such as spatial distribution and extent of infiltrates, overall cellularity and fibrosis. This also implies that trephine biopsy may be more useful in postchemotherapy patients to assess the residual tumor cell burden and degree of chemotherapy response. Newer techniques like flow cytometry can increase the sensitivity BMA in NHL patients, but could not be evaluated in the present study. Availability of broad panel of antibodies suitable for paraffin-embedded tissues, enables us to perform complete immunophenotyping on trephines and allows classification of lymphoma infiltrates according to established algorithms.^4^

Our finding that only 5% aspirates were positive, confirm the fact that BMA does not have much role in detecting marrow involvement by Hodgkin disease. Our findings are in agreement with those of Moid and Sharma et al.^17^,^20^ Although role of biopsy is controversial especially in stage I and IIA Hodgkin lymphoma, it is still irreplaceable in staging (especially in stage IIB or III cases) and hence alters the treatment.^21^ We recommend that instead of BMA, trephine biopsy should be done for staging in Hodgkin lymphoma. Necrosis is usually seen post chemotherapy, but we found very high incidence of necrosis and fibrosis (60%) at the time of primary diagnosis. Foci of fibrosis in the absence of classical or variant RS cells, with Hodgkin lymphoma diagnosed elsewhere, are highly suspicious of marrow involvement.^22^

BMA was 88.5% sensitive in diagnosis of multiple myeloma. Trephine biopsy helped to identify focal compact masses of plasma cells without any stroma in 7.7% patients which were missed on aspirate. Biopsy is more sensitive method for quantifying plasma cell burden (using CD138 IHC), especially in patients with low percentage of plasma cells on aspirate.^23^ However, cytomorphological classification of myeloma is better done on aspirate or imprint (mature, intermediary, immature and plasmablastic types).^24^

In acute leukemia, aspirate had a high accuracy of 91.2%. 10.6% aspirates were inadequate in which trephine biopsy showed near total replacement of marrow by blasts or myeloid precursors and extensive fibrosis. In MDS, aspirate was 100% sensitive but trephine biopsy provided additional information such as detection abnormal localization of immature precursors (ALIP) and aggregates of myeloblasts. Presence of fibrosis or fatty changes in marrow can make accurate disease characterization very difficult or impossible on aspirates.^25^ Literature suggests the utility of imprint cytology in providing excellent cytomorphological details in cases of dry tap,^7^ but we did not evaluate its role in the present study.

Peripheral smear and BMA may show overlapping findings in CMPNs. Role of trephine biopsy is not only in differentiation of CMPNs, but also to assess the overall marrow cellularity, histotopography and morphology of megakaryocytes and blasts (CD34 positive precursors) and degree of myelofibrosis.^26^ Non diagnostic aspirates in CML patients, who had grade 2 marrow fibrosis highlights the importance of trephine biopsy in CML. Also, focal collection of blasts occupying significant intertrabecular space in biopsy clinched the diagnosis of blast crises, irrespective of blast count in peripheral smear and BMA as was seen in our case.^26^

BMA does not have much role in diagnosis of PMF because diffuse osteomyelosclerosis, intrasinusoidal hematopoiesis and vascular proliferation, which are characteristic of fibrotic PMF, can be confirmed and graded on biopsy sections only.^27^

Megaloblastic anemia was the most common (37%) cause of pancytopenia in our study. High incidence of megaloblastic anemia can be explained by prevalent malnutrition and infectious diseases, seen in tropical country as ours. Aplastic anemia was the etiology in 19%, but aspirate was suggestive in only 38.5% cases. Trephine biopsy gives the qualitative and quantitative assessment of cellularity, therefore, is confirmatory in the diagnosis of aplastic anemia and overcomes the limitation of dry tap.^28^ In addition, biopsy can provide the number and distribution of megakaryocytes, lymphocytes, plasma cells in marrow and blasts, all of which are prognostic markers required in follow up of aplastic anemia.^28^

**Figure 9:**
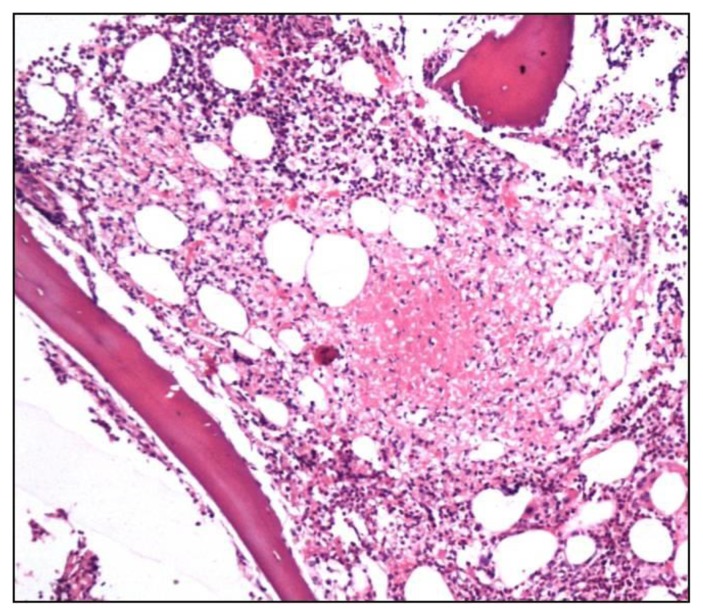
Trephine biopsy shows epithelioid cell granuloma with central necrosis (H&Ex100)

Rarely, Hodgkin and NHL can present as pan or bicytopenia without any evidence of lymphadenopathy/ hepatosplenomegaly as was seen in 5.9% cases.^22^ Disseminated tuberculosis was another important cause of pancytopenia in our population (5.9%). Aspirate is not useful in diagnosis of granulomatous myelitis as seen in our study, confirming the findings of Toi et al.^1^ Granulomatous response can be seen in tuberculosis, Hodgkin lymphoma, NHL, fungal infections and sarcoidosis.^29^ Though AFB stain was positive in 50% cases, presence of discrete epithelioid cell caseating granulomas and clinical findings were suggestive of disseminated tuberculosis in all of them (Figure 9). Very rarely, metastases can present with pancytopenia as was seen in neuroblastoma and small cell carcinoma (2.9% cases).

Amount of assessable marrow included in the biopsy specimen is of more importance than the total length. Logically, the likelihood of lymphoma positivity should rise concomitant with the length of interpretable biopsy specimen and examination of serial deeper sections. However, in our study no diagnostic gain was achieved above a length of 20 mm, with maximum percentage positivity (68.9%) obtained in biopsies 17–20 mm long. Trephine biopsies ≥17mm had significantly higher lymphoma positivity as compared to those of ≤16mm. The National Cancer Institute has recommended a trephine length of ≥20 mm for NHL staging.^11^ Campbell et al has supported this recommendation and emphasized the role of examining multiple sections.^12^ Bain suggested a minimum trephine length of 16 mm, based on the findings of Bishop set al. in which a plateau was achieved in the rate of detection of metastatic tumour after trephine length exceeded 16 mm.^30^,^31^ We found preprocessing trephine biopsy 17–20 mm long, along with examination of multiple deeper sections optimal for detection of lymphoma infiltration.

There are few limitations of our study. We did not evaluate the role of flow cytometry and touch imprint cytology in our study, both of which can increase the diagnostic accuracy. There were many subgroups in our study, some of which had small sample size. This is because we did not focus on any single disease, rather prospectively included all the patients presenting to us over a period of one year.

## Conclusion

Bone marrow aspirate is a simple and rapid alternative to biopsy, and has high specificity and positive predictive value. Aspirate is especially useful for acute leukemia, multiple myeloma, nutritional anemia, immune thrombocytopenias and other diffuse marrow disorders, where sensitivity and NPV are also equally good. Aspirate has a very limited role as far as Hodgkin lymphoma, granulomatous myelitis, aplastic anemia and myelofibrosis are concerned, making biopsy mandatory. In NHL, metastases and CMPNs, aspirate alone is insufficient and biopsy is complementary. Biopsy provides additional information like marrow fibrosis, pattern of marrow involvement, topographical alterations of hematopoietic cells, and postchemotherapy changes, which are prognostically useful. Trephine biopsies 17–20 mm long, examined at multiple deeper levels, had maximum proportion of lymphoma positivity and are optimal for assessing lymphoma infiltration. Biopsies longer than 20 mm don’t offer any added advantage.

## Figures and Tables

**Table 1:** Distribution of 382 patients of hematological malignancies and proportion of bone marrow positivity

Provisional Diagnosis	Number	Positive (%)	Negative
**Non Hodgkin Lymphoma**	133	67 (50.4)	66
**Hodgkin lymphoma**	47	20 (42.5)	27
CMPN*	93	81 (87.1)	12
**Acute leukemia**	54	44 (81.5)	10
**Multiple myeloma**	31	26 (83.8)	5
**Metastases**	23	10 (43.5)	13

* CMPN-Chronic myeloproliferative neoplasm.

**Table 2:** Concordance between bone marrow aspirate and biopsy

	Positive Biopsy	BMA Not Consistent	BMA Consistent	BMA Inadequate	Concordance rate (%)
**Non Hodgkin Lymphoma**	71	17	48	6	67.6
**Hodgkin lymphoma**	20	17	1	2	5.0
**Metastatic solid tumors**	12	3	7	2	58.3
**Multiple myeloma**	26	2	23	1	88.5
**CML CP**#**(grade 0–1 fibrosis)**	28	0	26	2	92.8
**CML CP**#**(Grade 2 fibrosis)**	30	0	23	7	76.7
**CML- Blast Phase**	12	2	10	0	83.3
**PMF**##	9	2	0	7	0.0
**Hypereosinophilic syndrome**	2	0	2	0	100
**Acute leukemia**	47	0	42	5	89.4
MDS*	2	0	2	0	100
**Nutritional/ Megaloblastic anemia**	25	0	24	1	96.0
**Aplastic anemia**	13	2	5	6	38.5
**Granulomatous inflammation**	4	3	0	1	0.0
**Leishmaniasis**	1	0	1	0	100
ITP**	2	0	2	0	100
**Hemophagocytic syndrome**	1	0	1	0	100
**Total**	305	46	219	40	71.8

# CML-Chronic myeloid leukemia- chronic phase,

## PMF-Primary myelofibrosis,

* MDS-Myelodysplastic syndrome,

** ITP-Immune thrombocytopenic purpura

**Table 3:** Validity parameters for bone marrow aspirate

	Sensitivity	Specificity	NPV*	PPV**	Accuracy
**NHL**	67.7	100	80.3	100	77.2
**Hodgkin**	5.0	100	58.7	100	59.6
**Metastases**	58.3	100	72.2	100	80
**Multiple myeloma**	88.5	100	62.5	100	90.3
**Acute leukemia**	89.4	100	60.7	100	91.2
**CMPN**#	75.3	100	37.5	100	78.5

# CMPN- Chronic myeloproliferative neoplasm,

* NPV- Negative predictive value,

** PPV-Positive predictive value

**Table 4:** Correlation of lymphoma positivity with trephine biopsy length

Length of trephine biopsy	Total no. of cases	Positive for Lymphoma infiltration	Negative for Lymphoma infiltration	Percentage positivity (%)
**1–4 mm**	8	2	6	25
**5–8 mm**	37	12	25	32.4
**9–12 mm**	54	23	31	42.6
**13–16 mm**	20	11	9	55.0
**17–20 mm**	29	20	9	68.9
**21–24 mm**	14	9	5	64.3
**25–28 mm**	11	7	4	63.6
**29–32 mm**	11	7	4	63.6
**Total**	184	91	93	49.4
